# (*E*)-*N*′-(3,4,5-Trimethoxy­benzyl­idene)-2-(8-quinol­yloxy)acetohydrazide methanol solvate

**DOI:** 10.1107/S1600536809051113

**Published:** 2009-12-04

**Authors:** Ling Zeng

**Affiliations:** a College of Chemistry and Chemical Engineering of Bohai University, Jinzhou, Liaoning 121000, People’s Republic of China

## Abstract

In the title compound, C_21_H_21_N_3_O_5_·CH_4_O, the quinoline plane and the benzene ring form a dihedral angle of 3.6 (2)°. The methanol solvent mol­ecule is linked with the acetohydrazide mol­ecule *via* O—H⋯N and N—H⋯O hydrogen bonds. In the crystal structure, weak inter­molecular C—H⋯O hydrogen bonds help to consolidate the crystal packing, which also exhibits π–π inter­actions, as indicated by short distances of 3.739 (4) Å between the centroids of the aromatic rings.

## Related literature

For applications of 8-hydroxy­quinoline derivatives, see: Park *et al.* (2006 ▶); Karmakar *et al.* (2007 ▶). For a related structure, see Wang *et al.* (2009 ▶).
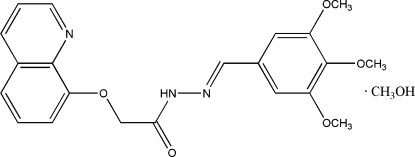


## Experimental

### 

#### Crystal data

C_21_H_21_N_3_O_5_·CH_4_O
*M* *_r_* = 427.45
Orthorhombic, 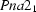

*a* = 13.385 (4) Å
*b* = 4.9005 (15) Å
*c* = 31.89 (1) Å
*V* = 2091.8 (11) Å^3^
*Z* = 4
Mo *K*α radiation
μ = 0.10 mm^−1^
*T* = 295 K
0.18 × 0.15 × 0.12 mm

#### Data collection

Bruker SMART CCD area-detector diffractometer
Absorption correction: multi-scan (*SADABS*; Sheldrick, 1996 ▶) *T*_min_ = 0.982, *T*_max_ = 0.988
10056 measured reflections
1879 independent reflections
1263 reflections with *I* > 2σ(*I*)
*R*_int_ = 0.077

#### Refinement

*R*[*F* ^2^ > 2σ(*F* ^2^)] = 0.043
*wR*(*F* ^2^) = 0.099
*S* = 1.08
1879 reflections
283 parameters
H-atom parameters constrained
Δρ_max_ = 0.15 e Å^−3^
Δρ_min_ = −0.16 e Å^−3^

### 

Data collection: *SMART* (Siemens, 1996 ▶); cell refinement: *SAINT* (Siemens, 1996 ▶); data reduction: *SAINT*; program(s) used to solve structure: *SHELXS97* (Sheldrick, 2008 ▶); program(s) used to refine structure: *SHELXL97* (Sheldrick, 2008 ▶); molecular graphics: *SHELXTL* (Sheldrick, 2008 ▶); software used to prepare material for publication: *SHELXTL*.

## Supplementary Material

Crystal structure: contains datablocks global, I. DOI: 10.1107/S1600536809051113/cv2668sup1.cif

Structure factors: contains datablocks I. DOI: 10.1107/S1600536809051113/cv2668Isup2.hkl

Additional supplementary materials:  crystallographic information; 3D view; checkCIF report

## Figures and Tables

**Table 1:** Hydrogen-bond geometry (Å, °)

*D*—H⋯*A*	*D*—H	H⋯*A*	*D*⋯*A*	*D*—H⋯*A*
O6—H6⋯N1	0.82	2.02	2.814 (5)	164
N2—H4⋯O6	0.86	2.30	3.070 (4)	149
C3—H3⋯O5^i^	0.93	2.45	3.340 (6)	159
C5—H5⋯O4^i^	0.93	2.52	3.411 (6)	160
C19—H19*A*⋯O2^ii^	0.96	2.37	3.196 (6)	144
C21—H21*A*⋯O3^iii^	0.96	2.57	3.265 (5)	130

Symmetry codes: (i) 
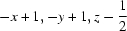
; (ii) 
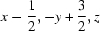
; (iii) 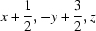.

**Fig. 1.**
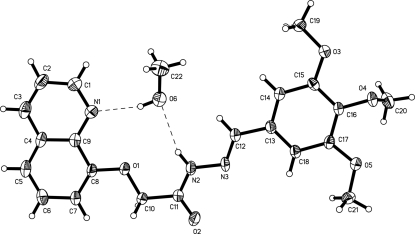
The molecular structure of (I), with displacement ellipsoids drawn at the 30% probability level. Dashed lines indicate hydrogen bonds.

## supplementary crystallographic information

### Comment

Synthesis of 8-hydroxyquinoline and its derivatives have attracted a great interest due to their biological activities and applications in coordination chemistry (Park *et al.*, 2006; Karmakar *et al.*, 2007). In our search for new extractants of metal ions and biologically active materials, the title compound, (I), has been synthesized. We report here its crystal structure.

All bond lengths and angles are normal and comparable to those observed in the related compound (*E*)-*N'*-(2,5-dimethoxybenzylidene)-2-(8- quinolyloxy)acetohydrazide methanol solvate (Wang *et al.*, 2009). The molecule is nearly planar, with a dihedral angle of the benzene ring and the quinoline ring of 3.6 (2)°. The methanol solvent molecule forms an O—H···N hydrogen bond to the quinoline ring system and accepts an N—H···O hydrogen bond from the hydrazide NH group. In the crystal structure, weak intermolecular C—H···O hydrogen bonds (Table 1) help to consolidate the crystal packing.

### Experimental

3,4,5-Trimethoxybenzaldehyde (0.1 mmol, 19.6 mg) and 2-(quinolin-8-yloxy) acetohydrazide (21.8 mg, 0.1 mmol), were dissolved in methanol (20 ml). The mixture was stirred at room temperature to give a clear colorless solution. Crystals of the title compound were formed by gradual evaporation of the solvent over a period of six days at room temperature.

### Refinement

All H atoms were initially located in a difference Fourier map, then placed in idealized positions (C—H  0.93–0.97 Å, O—H  0.82–0.85 Å, N—H  0.86 Å) and refined as riding, with *U*_iso_(H) = 1.2*U*_eq_(C, N) and 1.5*U*_eq_(O). In the absence of atoms heavier than Si, the absolute structure can not be reliably determined, so 1784 Friedel pairs were averaged before the final refinement.

### Crystal data

**Table:**

C_21_H_21_N_3_O_5_·CH_4_O	*F*(000) = 904
*M**_r_* = 427.45	*D*_x_ = 1.357 Mg m^−^^3^
Orthorhombic, *P**n**a*2_1_	Mo *K*α radiation, λ = 0.71073 Å
Hall symbol: P 2c -2n	Cell parameters from 786 reflections
*a* = 13.385 (4) Å	θ = 2.6–17.8°
*b* = 4.9005 (15) Å	µ = 0.10 mm^−^^1^
*c* = 31.89 (1) Å	*T* = 295 K
*V* = 2091.8 (11) Å^3^	Block, colourless
*Z* = 4	0.18 × 0.15 × 0.12 mm

### Data collection

**Table:**

Bruker SMART CCD area-detector diffractometer	1879 independent reflections
Radiation source: fine-focus sealed tube	1263 reflections with *I* > 2σ(*I*)
graphite	*R*_int_ = 0.077
φ and ω scans	θ_max_ = 25.1°, θ_min_ = 2.6°
Absorption correction: multi-scan (*SADABS*; Sheldrick, 1996)	*h* = −15→13
*T*_min_ = 0.982, *T*_max_ = 0.988	*k* = −5→5
10056 measured reflections	*l* = −37→37

### Refinement

**Table:**

Refinement on *F*^2^	Secondary atom site location: difference Fourier map
Least-squares matrix: full	Hydrogen site location: inferred from neighbouring sites
*R*[*F*^2^ > 2σ(*F*^2^)] = 0.043	H-atom parameters constrained
*wR*(*F*^2^) = 0.099	*w* = 1/[σ^2^(*F*_o_^2^) + (0.0351*P*)^2^ + 0.0119*P*] where *P* = (*F*_o_^2^ + 2*F*_c_^2^)/3
*S* = 1.08	(Δ/σ)_max_ < 0.001
1879 reflections	Δρ_max_ = 0.15 e Å^−^^3^
283 parameters	Δρ_min_ = −0.16 e Å^−^^3^
0 restraints	Extinction correction: *SHELXL97* (Sheldrick, 2008), Fc^*^=kFc[1+0.001xFc^2^λ^3^/sin(2θ)]^-1/4^
Primary atom site location: structure-invariant direct methods	Extinction coefficient: 0.0111 (15)

### Special details

**Table:**

Geometry. All e.s.d.'s (except the e.s.d. in the dihedral angle between two l.s. planes) are estimated using the full covariance matrix. The cell e.s.d.'s are taken into account individually in the estimation of e.s.d.'s in distances, angles and torsion angles; correlations between e.s.d.'s in cell parameters are only used when they are defined by crystal symmetry. An approximate (isotropic) treatment of cell e.s.d.'s is used for estimating e.s.d.'s involving l.s. planes.
Refinement. Refinement of *F*^2^ against ALL reflections. The weighted *R*-factor *wR* and goodness of fit *S* are based on *F*^2^, conventional *R*-factors *R* are based on *F*, with *F* set to zero for negative *F*^2^. The threshold expression of *F*^2^ > σ(*F*^2^) is used only for calculating *R*-factors(gt) *etc*. and is not relevant to the choice of reflections for refinement. *R*-factors based on *F*^2^ are statistically about twice as large as those based on *F*, and *R*- factors based on ALL data will be even larger.

### Fractional atomic coordinates and isotropic or equivalent isotropic displacement parameters (Å^2^)

**Table:**

	*x*	*y*	*z*	*U*_iso_*/*U*_eq_	
O1	0.6229 (2)	0.7115 (6)	0.13448 (8)	0.0497 (8)	
O2	0.6522 (3)	1.1590 (8)	0.22093 (11)	0.0819 (12)	
O3	0.1718 (2)	0.3242 (6)	0.34960 (8)	0.0495 (8)	
O4	0.2323 (2)	0.6336 (6)	0.41318 (8)	0.0448 (8)	
O5	0.3852 (2)	0.9783 (6)	0.40569 (9)	0.0515 (9)	
O6	0.4246 (3)	0.4033 (8)	0.16176 (12)	0.0776 (12)	
H6	0.4642	0.3998	0.1421	0.116*	
N1	0.5266 (3)	0.3563 (9)	0.08495 (12)	0.0543 (11)	
N2	0.5580 (3)	0.7798 (8)	0.21461 (10)	0.0475 (10)	
H4	0.5427	0.6407	0.1995	0.057*	
N3	0.5127 (3)	0.8173 (8)	0.25337 (10)	0.0449 (10)	
C1	0.4809 (4)	0.1830 (12)	0.06048 (17)	0.0679 (16)	
H1	0.4270	0.0872	0.0716	0.081*	
C2	0.5070 (5)	0.1317 (12)	0.01916 (17)	0.0679 (16)	
H2	0.4720	0.0042	0.0033	0.082*	
C3	0.5852 (4)	0.2727 (12)	0.00229 (15)	0.0635 (15)	
H3	0.6037	0.2437	−0.0255	0.076*	
C4	0.6372 (4)	0.4592 (10)	0.02669 (14)	0.0481 (12)	
C5	0.7184 (4)	0.6099 (12)	0.01122 (15)	0.0610 (15)	
H5	0.7386	0.5885	−0.0165	0.073*	
C6	0.7676 (4)	0.7867 (11)	0.03669 (15)	0.0597 (14)	
H6A	0.8220	0.8836	0.0263	0.072*	
C7	0.7376 (4)	0.8255 (11)	0.07854 (14)	0.0530 (13)	
H7	0.7724	0.9470	0.0955	0.064*	
C8	0.6581 (3)	0.6872 (10)	0.09433 (12)	0.0436 (12)	
C9	0.6056 (3)	0.4985 (10)	0.06885 (13)	0.0439 (12)	
C10	0.6703 (3)	0.9191 (9)	0.15890 (14)	0.0486 (12)	
H10A	0.6671	1.0901	0.1436	0.058*	
H10B	0.7402	0.8724	0.1624	0.058*	
C11	0.6250 (3)	0.9590 (11)	0.20091 (14)	0.0479 (12)	
C12	0.4417 (3)	0.6516 (10)	0.26137 (14)	0.0463 (12)	
H12	0.4241	0.5212	0.2415	0.056*	
C13	0.3880 (3)	0.6625 (10)	0.30083 (12)	0.0417 (12)	
C14	0.3068 (3)	0.4905 (9)	0.30537 (12)	0.0421 (11)	
H14	0.2883	0.3768	0.2833	0.050*	
C15	0.2527 (3)	0.4860 (9)	0.34250 (12)	0.0389 (11)	
C16	0.2808 (3)	0.6550 (10)	0.37560 (12)	0.0374 (11)	
C17	0.3629 (3)	0.8264 (9)	0.37111 (12)	0.0405 (11)	
C18	0.4170 (3)	0.8321 (9)	0.33362 (12)	0.0409 (12)	
H18	0.4717	0.9477	0.3306	0.049*	
C19	0.1410 (4)	0.1460 (10)	0.31644 (14)	0.0520 (12)	
H19A	0.1206	0.2525	0.2927	0.078*	
H19B	0.0861	0.0359	0.3258	0.078*	
H19C	0.1957	0.0301	0.3086	0.078*	
C20	0.1689 (4)	0.8559 (10)	0.42331 (16)	0.0624 (15)	
H20A	0.2052	1.0238	0.4203	0.094*	
H20B	0.1462	0.8378	0.4517	0.094*	
H20C	0.1125	0.8567	0.4047	0.094*	
C21	0.4632 (3)	1.1764 (10)	0.40269 (14)	0.0513 (13)	
H21A	0.5264	1.0852	0.4002	0.077*	
H21B	0.4633	1.2881	0.4274	0.077*	
H21C	0.4524	1.2889	0.3785	0.077*	
C22	0.3780 (4)	0.1473 (12)	0.1656 (2)	0.0755 (16)	
H22A	0.4269	0.0056	0.1624	0.113*	
H22B	0.3280	0.1286	0.1442	0.113*	
H22C	0.3472	0.1329	0.1927	0.113*	

### Atomic displacement parameters (Å^2^)

**Table:**

	*U*^11^	*U*^22^	*U*^33^	*U*^12^	*U*^13^	*U*^23^
O1	0.0574 (19)	0.061 (2)	0.0310 (16)	−0.0138 (17)	0.0085 (14)	−0.0099 (15)
O2	0.104 (3)	0.092 (3)	0.050 (2)	−0.048 (2)	0.0258 (19)	−0.030 (2)
O3	0.052 (2)	0.058 (2)	0.0388 (17)	−0.0123 (18)	0.0051 (14)	−0.0016 (16)
O4	0.0519 (19)	0.052 (2)	0.0299 (16)	0.0046 (16)	0.0083 (14)	0.0080 (15)
O5	0.0585 (19)	0.061 (2)	0.0350 (17)	−0.0140 (19)	0.0038 (15)	−0.0064 (16)
O6	0.088 (3)	0.080 (3)	0.064 (3)	−0.023 (2)	0.018 (2)	−0.018 (2)
N1	0.056 (2)	0.059 (3)	0.048 (2)	−0.009 (2)	0.000 (2)	−0.008 (2)
N2	0.058 (2)	0.054 (3)	0.031 (2)	−0.001 (2)	0.0097 (17)	−0.0052 (18)
N3	0.051 (2)	0.057 (3)	0.0264 (19)	0.003 (2)	0.0121 (18)	−0.0002 (18)
C1	0.063 (3)	0.080 (4)	0.060 (3)	−0.016 (3)	0.000 (3)	−0.019 (3)
C2	0.080 (4)	0.073 (4)	0.052 (3)	−0.001 (4)	−0.011 (3)	−0.022 (3)
C3	0.077 (4)	0.076 (4)	0.037 (3)	0.017 (3)	−0.007 (3)	−0.010 (3)
C4	0.064 (3)	0.048 (3)	0.033 (3)	0.014 (3)	−0.001 (2)	−0.005 (2)
C5	0.077 (4)	0.070 (4)	0.036 (3)	0.013 (3)	0.010 (3)	−0.007 (3)
C6	0.067 (3)	0.069 (4)	0.043 (3)	0.004 (3)	0.017 (3)	0.002 (3)
C7	0.058 (3)	0.065 (4)	0.036 (3)	−0.004 (3)	0.012 (2)	−0.008 (2)
C8	0.052 (3)	0.052 (3)	0.027 (2)	−0.001 (3)	0.005 (2)	−0.003 (2)
C9	0.051 (3)	0.043 (3)	0.038 (3)	0.007 (3)	0.002 (2)	0.000 (2)
C10	0.052 (3)	0.060 (3)	0.034 (2)	−0.013 (3)	0.005 (2)	−0.010 (2)
C11	0.046 (3)	0.062 (4)	0.035 (3)	−0.010 (3)	0.006 (2)	−0.009 (3)
C12	0.051 (3)	0.055 (3)	0.033 (2)	−0.005 (3)	0.008 (2)	−0.003 (2)
C13	0.041 (3)	0.052 (3)	0.032 (2)	0.002 (2)	0.005 (2)	0.001 (2)
C14	0.047 (3)	0.047 (3)	0.033 (2)	−0.001 (2)	0.0023 (19)	−0.003 (2)
C15	0.037 (3)	0.043 (3)	0.037 (3)	0.001 (2)	0.0049 (19)	0.010 (2)
C16	0.041 (3)	0.042 (3)	0.028 (2)	0.003 (2)	0.0052 (19)	0.002 (2)
C17	0.046 (3)	0.047 (3)	0.029 (2)	0.007 (2)	−0.0021 (19)	0.001 (2)
C18	0.043 (3)	0.047 (3)	0.032 (2)	0.001 (2)	0.004 (2)	0.004 (2)
C19	0.057 (3)	0.058 (3)	0.041 (3)	−0.015 (3)	−0.005 (2)	−0.001 (3)
C20	0.070 (3)	0.058 (4)	0.059 (3)	0.002 (3)	0.025 (3)	0.002 (3)
C21	0.050 (3)	0.050 (3)	0.053 (3)	−0.007 (3)	−0.003 (2)	−0.010 (2)
C22	0.069 (4)	0.069 (4)	0.088 (4)	−0.001 (3)	−0.003 (3)	0.008 (3)

### Geometric parameters (Å, °)

**Table:**

O1—C8	1.369 (5)	C7—C8	1.358 (6)
O1—C10	1.429 (5)	C7—H7	0.9300
O2—C11	1.225 (5)	C8—C9	1.418 (6)
O3—C15	1.360 (5)	C10—C11	1.483 (6)
O3—C19	1.432 (5)	C10—H10A	0.9700
O4—C16	1.367 (5)	C10—H10B	0.9700
O4—C20	1.418 (5)	C12—C13	1.450 (6)
O5—C17	1.364 (5)	C12—H12	0.9300
O5—C21	1.429 (5)	C13—C14	1.382 (6)
O6—C22	1.407 (6)	C13—C18	1.391 (6)
O6—H6	0.8200	C14—C15	1.389 (5)
N1—C1	1.306 (6)	C14—H14	0.9300
N1—C9	1.366 (6)	C15—C16	1.394 (6)
N2—C11	1.329 (5)	C16—C17	1.390 (6)
N2—N3	1.389 (5)	C17—C18	1.398 (6)
N2—H4	0.8600	C18—H18	0.9300
N3—C12	1.275 (5)	C19—H19A	0.9600
C1—C2	1.386 (7)	C19—H19B	0.9600
C1—H1	0.9300	C19—H19C	0.9600
C2—C3	1.365 (8)	C20—H20A	0.9600
C2—H2	0.9300	C20—H20B	0.9600
C3—C4	1.387 (7)	C20—H20C	0.9600
C3—H3	0.9300	C21—H21A	0.9600
C4—C5	1.404 (7)	C21—H21B	0.9600
C4—C9	1.422 (6)	C21—H21C	0.9600
C5—C6	1.358 (7)	C22—H22A	0.9600
C5—H5	0.9300	C22—H22B	0.9600
C6—C7	1.407 (6)	C22—H22C	0.9600
C6—H6A	0.9300		
			
C8—O1—C10	114.8 (3)	N3—C12—C13	121.3 (4)
C15—O3—C19	117.5 (3)	N3—C12—H12	119.4
C16—O4—C20	115.1 (3)	C13—C12—H12	119.4
C17—O5—C21	118.5 (3)	C14—C13—C18	120.3 (4)
C22—O6—H6	109.5	C14—C13—C12	117.3 (4)
C1—N1—C9	118.0 (4)	C18—C13—C12	122.4 (4)
C11—N2—N3	120.0 (4)	C13—C14—C15	120.6 (4)
C11—N2—H4	120.0	C13—C14—H14	119.7
N3—N2—H4	120.0	C15—C14—H14	119.7
C12—N3—N2	114.8 (4)	O3—C15—C14	124.5 (4)
N1—C1—C2	124.6 (5)	O3—C15—C16	115.8 (4)
N1—C1—H1	117.7	C14—C15—C16	119.7 (4)
C2—C1—H1	117.7	O4—C16—C17	120.8 (4)
C3—C2—C1	118.4 (5)	O4—C16—C15	119.3 (4)
C3—C2—H2	120.8	C17—C16—C15	119.6 (3)
C1—C2—H2	120.8	O5—C17—C16	114.8 (3)
C2—C3—C4	119.8 (5)	O5—C17—C18	124.5 (4)
C2—C3—H3	120.1	C16—C17—C18	120.6 (4)
C4—C3—H3	120.1	C13—C18—C17	119.1 (4)
C3—C4—C5	122.5 (5)	C13—C18—H18	120.5
C3—C4—C9	118.1 (5)	C17—C18—H18	120.5
C5—C4—C9	119.4 (5)	O3—C19—H19A	109.5
C6—C5—C4	120.1 (5)	O3—C19—H19B	109.5
C6—C5—H5	120.0	H19A—C19—H19B	109.5
C4—C5—H5	120.0	O3—C19—H19C	109.5
C5—C6—C7	121.0 (5)	H19A—C19—H19C	109.5
C5—C6—H6A	119.5	H19B—C19—H19C	109.5
C7—C6—H6A	119.5	O4—C20—H20A	109.5
C8—C7—C6	120.6 (5)	O4—C20—H20B	109.5
C8—C7—H7	119.7	H20A—C20—H20B	109.5
C6—C7—H7	119.7	O4—C20—H20C	109.5
C7—C8—O1	124.9 (4)	H20A—C20—H20C	109.5
C7—C8—C9	120.1 (4)	H20B—C20—H20C	109.5
O1—C8—C9	115.0 (4)	O5—C21—H21A	109.5
N1—C9—C8	120.1 (4)	O5—C21—H21B	109.5
N1—C9—C4	121.1 (4)	H21A—C21—H21B	109.5
C8—C9—C4	118.9 (4)	O5—C21—H21C	109.5
O1—C10—C11	113.9 (4)	H21A—C21—H21C	109.5
O1—C10—H10A	108.8	H21B—C21—H21C	109.5
C11—C10—H10A	108.8	O6—C22—H22A	109.5
O1—C10—H10B	108.8	O6—C22—H22B	109.5
C11—C10—H10B	108.8	H22A—C22—H22B	109.5
H10A—C10—H10B	107.7	O6—C22—H22C	109.5
O2—C11—N2	123.9 (4)	H22A—C22—H22C	109.5
O2—C11—C10	117.0 (4)	H22B—C22—H22C	109.5
N2—C11—C10	119.1 (4)		
			
C11—N2—N3—C12	172.4 (4)	O1—C10—C11—O2	−169.6 (4)
C9—N1—C1—C2	0.2 (8)	O1—C10—C11—N2	10.2 (7)
N1—C1—C2—C3	−0.7 (9)	N2—N3—C12—C13	179.2 (4)
C1—C2—C3—C4	0.8 (8)	N3—C12—C13—C14	174.7 (4)
C2—C3—C4—C5	179.8 (5)	N3—C12—C13—C18	−7.0 (7)
C2—C3—C4—C9	−0.6 (7)	C18—C13—C14—C15	0.5 (7)
C3—C4—C5—C6	−179.0 (5)	C12—C13—C14—C15	178.9 (4)
C9—C4—C5—C6	1.5 (7)	C19—O3—C15—C14	0.2 (6)
C4—C5—C6—C7	−1.0 (8)	C19—O3—C15—C16	−179.5 (4)
C5—C6—C7—C8	−0.3 (8)	C13—C14—C15—O3	179.8 (4)
C6—C7—C8—O1	−179.7 (4)	C13—C14—C15—C16	−0.4 (6)
C6—C7—C8—C9	1.0 (7)	C20—O4—C16—C17	76.2 (5)
C10—O1—C8—C7	6.0 (6)	C20—O4—C16—C15	−109.2 (5)
C10—O1—C8—C9	−174.7 (4)	O3—C15—C16—O4	5.0 (6)
C1—N1—C9—C8	−179.4 (5)	C14—C15—C16—O4	−174.7 (4)
C1—N1—C9—C4	0.0 (7)	O3—C15—C16—C17	179.7 (4)
C7—C8—C9—N1	179.0 (4)	C14—C15—C16—C17	0.0 (6)
O1—C8—C9—N1	−0.3 (6)	C21—O5—C17—C16	−174.9 (4)
C7—C8—C9—C4	−0.5 (6)	C21—O5—C17—C18	6.1 (6)
O1—C8—C9—C4	−179.8 (4)	O4—C16—C17—O5	−4.0 (6)
C3—C4—C9—N1	0.2 (6)	C15—C16—C17—O5	−178.6 (4)
C5—C4—C9—N1	179.7 (5)	O4—C16—C17—C18	175.1 (4)
C3—C4—C9—C8	179.7 (4)	C15—C16—C17—C18	0.5 (6)
C5—C4—C9—C8	−0.8 (6)	C14—C13—C18—C17	−0.1 (7)
C8—O1—C10—C11	174.8 (4)	C12—C13—C18—C17	−178.4 (4)
N3—N2—C11—O2	1.5 (7)	O5—C17—C18—C13	178.6 (4)
N3—N2—C11—C10	−178.3 (4)	C16—C17—C18—C13	−0.4 (6)

### Hydrogen-bond geometry (Å, °)

**Table:**

*D*—H···*A*	*D*—H	H···*A*	*D*···*A*	*D*—H···*A*
O6—H6···N1	0.82	2.02	2.814 (5)	164
N2—H4···O6	0.86	2.30	3.070 (4)	149
C3—H3···O5^i^	0.93	2.45	3.340 (6)	159
C5—H5···O4^i^	0.93	2.52	3.411 (6)	160
C19—H19A···O2^ii^	0.96	2.37	3.196 (6)	144
C21—H21A···O3^iii^	0.96	2.57	3.265 (5)	130

Symmetry codes: (i) −*x*+1, −*y*+1, *z*−1/2; (ii) *x*−1/2, −*y*+3/2, *z*; (iii) *x*+1/2, −*y*+3/2, *z*.
